# Measuring coverage of essential maternal and newborn care interventions: An unfinished agenda to define the data matrix for action in maternal and newborn health

**DOI:** 10.7189/07.020307

**Published:** 2017-12

**Authors:** Allisyn Moran, Tanya Marchant

**Affiliations:** 1 Department of Maternal, Newborn, Child and Adolescent Health, World Health Organization, Geneva, Switzerland; 2 Department of Disease Control, London School of Hygiene and Tropical Medicine, London, UK

This collection provides crucial evidence on progress made and outstanding challenges on the road to improving maternal and newborn health using national household data (Demographic and Health Surveys; Multiple Indicator Cluster Surveys) and facility data (Service Provision Assessment) on multi-country coverage of maternal and newborn care seeking and care provision. Of the 11 manuscripts in this collection, six point to the need for more high quality, respectful care provided by health professionals working in enabling environments. Here we consider which data are fit for this improvement purpose.

## THE MATERNAL AND NEWBORN MEASUREMENT DYAD

The Sustainable Development Goals have aided the alignment of global strategies across reproductive, maternal, newborn, child and adolescent health. Central to this is recognition that in-service provision as well as measurement it is essential to keep the mother and baby together as a dyad, especially around the time of birth when the majority of maternal and newborn deaths occur. Despite the considerable progress by household and facility surveys to illuminate evidence on the content of care, robust data on quality life-saving care at birth remains scarce in many settings [1-3], and there continues to be a need for global guidance on best measurement methods.

## DATA FIT FOR THE PROGRAMMATIC CONTEXT

As practised by disease-specific initiatives such as UNAIDS [4], improving programmes for mothers and newborns requires a combination of data sources. Core indicators from national survey platforms are an essential part of the data matrix, but timely data from delivery rooms that can prospectively inform the decisions of health system actors at multiple levels are also needed. Inevitably this means that well-functioning health management information systems plus civil registration and vital statistics platforms are essential, especially when supported by innovations to summarise and visualize these data. Additional platforms may also be needed to provide more granular quality assessments, for example sentinel surveillance in communities and special studies in facilities.

**Figure:**
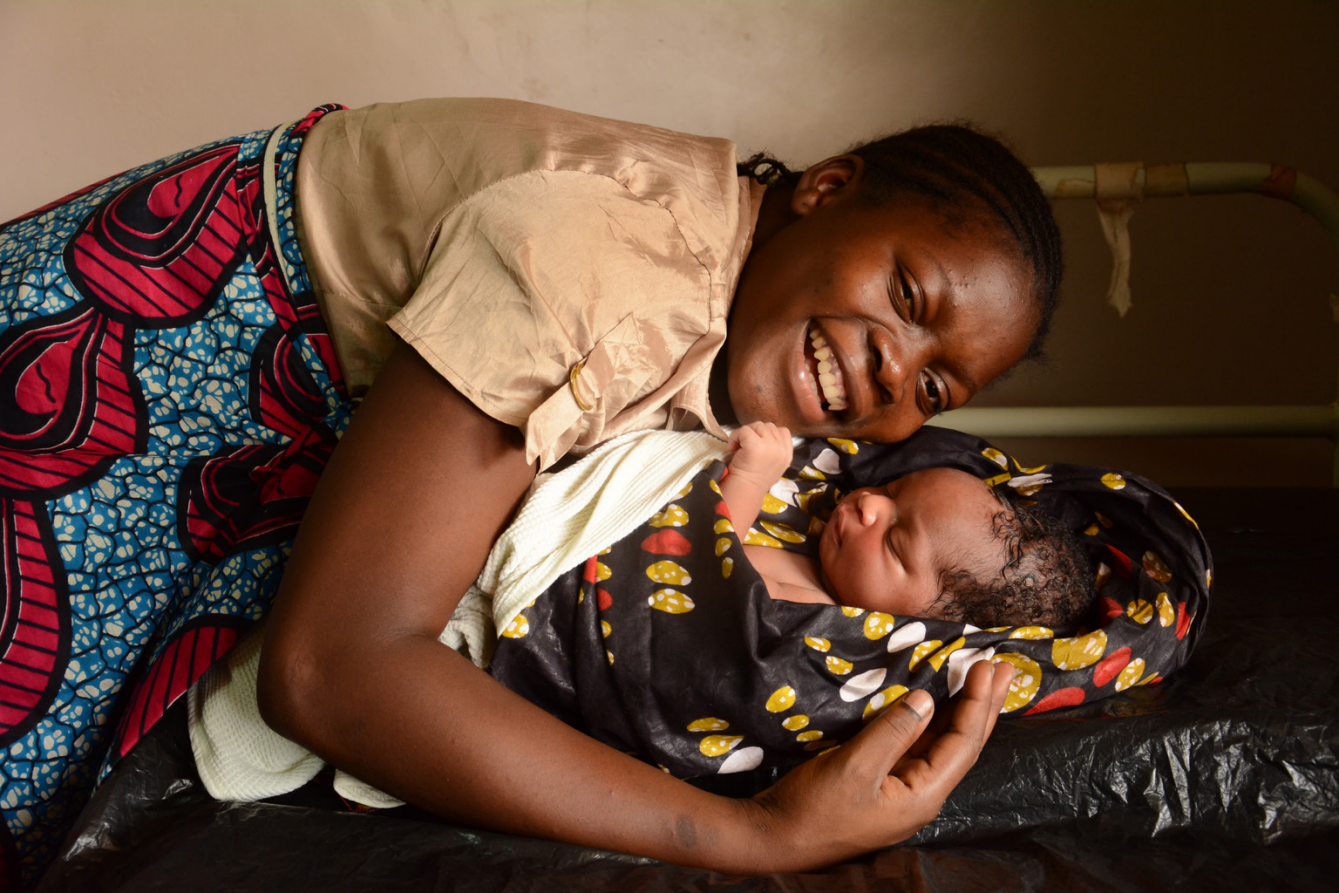
Photo: (c) UNICEF/UN018538/Chikondi (used with permission).

When optimized, these data sources in combination have powerful potential to advance the quality of maternal and newborn care. But defining a complex data matrix alone cannot remove the barrier that poor quality of care poses to maternal and newborn survival: careful guidance is needed to help actors prioritize and organize evidence for action. Considerable work has already been carried out to understand data needs and method limitations [5]. Work is under way to develop guidance on indicators and data collection tools for measurement of maternal and newborn programmes including suggestions for maximizing use of all data sources; however, as research is conducted the guidance will need to be updated and refined to reflect new recommendations. To further accelerate progress now the maternal and newborn health community must work to make sense of when and how each data source can be made to work together.

## References

[1] Moxon SG, Ruysen H, Kerber KJ, Amouzou A, Fourniers S, Grove J (2015). Count every newborn; a measurement improvement roadmap for coverage data.. BMC Pregnancy Childbirth.

[2] Marchant T, Bryce J, Victora C, Moran AC, Claeson M, Requejo J (2016). Improved measurement for mothers, newborns and children in the era of the Sustainable Development Goals.. J Glob Health.

[3] Munos MK, Stanton CK, Bryce J (2017). Core Group for Improving Coverage Measurement for MNCH. Improving coverage measurement for reproductive, maternal, neonatal and child health: gaps and opportunities.. J Glob Health.

[4] UNAIDS. Global AIDS Monitoring 2017: Indicators for monitoring the 2016 United Nations Political Declaration on HIV and AIDS. Available: http://www.unaids.org/sites/default/files/media_asset/2017-Global-AIDS-Monitoring_en.pdf. Accessed: 23 October 2017.

[5] World Health Organization. Mother and Newborn Information for Tracking Outcomes and Results (MONITOR) technical advisory group. Available: http://www.who.int/maternal child adolescent/epidemiology/monitor/en/. Accessed: 23 October 2017.

